# Helminth infection modulates number and function of adipose tissue Tregs in high fat diet-induced obesity

**DOI:** 10.1371/journal.pntd.0010105

**Published:** 2022-05-02

**Authors:** Camila P. Queiroz-Glauss, Mariana S. Vieira, Marcela Helena Gonçalves-Pereira, Stephanie S. Almeida, Rachel H. Freire, Maria A. Gomes, Jacqueline I. Alvarez-Leite, Helton C. Santiago

**Affiliations:** 1 Departamento de Bioquímica e Imunologia, Instituto de Ciências Biológicas, Universidade Federal de Minas Gerais, Belo Horizonte, Brazil; 2 Departamento de Parasitologia, Instituto de Ciências Biológicas, Universidade Federal de Minas Gerais, Belo Horizonte, Brazil; National University of Ireland Galway, IRELAND

## Abstract

**Background:**

Epidemiological and experimental studies have shown a protective effect of helminth infections in weight gain and against the development of metabolic dysfunctions in the host. However, the mechanisms Treg cells exert in the helminth-obesity interface has been poorly investigated. The present study aimed to verify the influence of *Heligmosomoides polygyrus* infection in early stages of high fat diet-induced obesity.

**Principal findings:**

The presence of infection was able to prevent exacerbated weight gain in mice fed with high fat diet when compared to non-infected controls. In addition, infected animals displayed improved insulin sensitivity and decreased fat accumulation in the liver. Obesity-associated inflammation was reduced in the presence of infection, demonstrated by lower levels of leptin and resistin, lower infiltration of Th1 and Th17 cells in adipose tissue, higher expression of IL10 and adiponectin, increased infiltration of Th2 and eosinophils in adipose tissue of infected animals. Of note, the parasite infection was associated with increased Treg frequency in adipose tissue which showed higher expression of cell surface markers of function and activation, like LAP and CD134. The infection could also increase adipose Treg suppressor function in animals on high fat diet.

**Conclusion:**

These data suggest that *H*. *polygyrus* modulates adipose tissue Treg cells with implication for weight gain and metabolic syndrome.

## Author summary

Helminth infections are known to modulate the immune system being responsible for protecting the host from developing allergic and autoimmune disorders (Hygiene Hypothesis). We hypothesized that the same immunomodulatory effect could have an impact on immunometabolic diseases, such as obesity and its linked diseases such as type 2 diabetes. Weight disorders have reached epidemic levels, nearly tripling since 1975 and being responsible for almost 5 million premature deaths each year, but have been spared in areas of high helminth prevalence. To test our hypothesis C57BL/6 male mice were fed control or high fat diet, for five weeks, in the presence or not of infection with the worm *Heligmosomoides polygyrus*. Weight gain, development of metabolic disorders, inflammation and cellular migration to the adipose tissue were evaluated. In accordance with our hypothesis, we found that the presence of infection prevented the exacerbated weight gain and also improved metabolic parameters in animals fed a high fat diet. This was associated with the infection’s ability to modulate parameters of a cell responsible for regulatory functions: Tregs. In the light of these findings, helminth infection could be protective against weight gain and metabolic disturbances.

## Introduction

Hygiene Hypothesis postulates that the stimulation of the immune system by microbes or microbial products, especially during childhood, can protect the host against the development of atopic and inflammatory disorders [1]. A number of epidemiological and experimental studies show a benefit effect of infections by viruses, bacteria, and helminths in the development of different inflammatory diseases like asthma [2–4], type 1 diabetes [5–8] and multiple sclerosis [9–10]. The positive influence of helminth infections in immunometabolic disorders, as obesity, has also been investigated [11–12].

Overweight and obesity are global health problems that reached epidemical status with more than 2 billion people with overweight and 650 million obese [13]. The greatest concern about this overnutrition syndrome is the association with the development of a number of serious metabolic consequences such as glucose metabolism dysfunctions [12], heart diseases [14] and even some types of cancer [15–22]. The establishment of these dysfunctions along with weight gain is linked to the development of a low-grade chronic inflammation [12]. For example, adipose tissue fat accumulation is associated with the migration of inflammatory cells like macrophages [23–24], mastocytes [25] and T cells [26] that produce pro-inflammatory cytokines, such as TNF and IL6, which contributes to dysfunctions in glucose metabolism [27]. Interestingly, some works have associated obesity with decreased frequency and dysfunction of adipose Treg cells [12,28], which is a possible mechanism to perpetuate background inflammation and metabolic syndrome. On the other hand, helminths are known to improve Treg function as a mechanism of immune evasion [29–31] and also in inflammatory disease models, such type 1 diabetes [25,32] and asthma [33,34]. Helminth infections have been shown to influence the weight gain and the development of metabolic dysfunctions [12] in experimental models of obesity [35–41] and in epidemiological studies in humans [42–45], but surprisingly, the role of Tregs in helminth-obesity interface has been poorly investigated.

The mechanisms mostly linked with helminth-associated control of weight gain are thought to be mediated by increased infiltration of eosinophils [40], Th2 cells [37], ILC2 [37], and decreased Th1 and Th17 profiles [44,46]. On the other hand, obesity is associated with Treg dysfunction [28,47] and experimental models have shown that Tregs are able to modulate obesity since their induction or transfer to obese animals can improve metabolic syndrome parameters and it is discussed to be used as therapeutic strategy [28,48,49].

In the current study, we examined the effect of infection with *H*. *polygyrus* on early stages of high fat diet (HFD)-induced obesity in mice and showed the ability of the infection to modulate the number, phenotype and function of adipose tissue Tregs cells, which may play a role in preventing weight gain and metabolic dysfunctions. Our results show that improvement of metabolic syndrome associated with obesity experimental model is paralleled to improvement in Treg numbers and function in adipose tissue.

## Methods

### Ethics statement

All the experiments were approved by the Ethics Committee for Animal Use of the Federal University of Minas Gerais (protocol#25/2012).

### Animals, diets and *H*. *polygyrus* infection

Male C57BL/6Unib mice, specific pathogen free, with four weeks of age were obtained at the Central Breeding Center of the Federal University of Minas Gerais. Throughout the experiment, animals were housed in temperature-controlled room with 12-hour light-dark cycle, with food and water available *ad libitum*. Mice were fed with high fat diet (HFD) (62% energy derived from fat, 23% from carbohydrates and 15% from proteins) or low fat diet (LFD) (10% energy derived from fat, 74% from carbohydrates and 16% from proteins) for five weeks. Caloric intake was measured every week, considering the weight difference between the amount of diet offered and the next week’s left overs, divided by the number of animals per cage. This value was then multiplied by the number of calories per gram of each diet (LFD: 2.76 kcal/g; HFD: 5.21 kcal/g). Concomitantly with the diet, experimental group received 200 L3 *H*. *polygyrus* larvae by oral gavage. Eggs in feces were detected by visual observation of feces smears under microscope to confirm successful patent infection.

### Insulin tolerance test and oral glucose tolerance test

The insulin tolerance test (ITT) was performed four days before euthanasia. Animals were bled at the tail vein and glucose levels were measured by a glucometer (Accu-Chek Performa; Roche, Diagnostics, USA) before, and 15, 30 and 60 minutes after 0.75 U/kg insulin injection i.p. Two days before euthanasia, oral glucose tolerance test (OGTT) was carried out after 6 hours of fasting. Mice were given glucose (2g/kg of body weight) by gavage and blood glucose levels were measured by a glucometer (Accu-Chek Performa; Roche) before, and 15, 30, 60 and 120 minutes after gavage.

### Nutrition parameters and lipid profile analysis

At the end of five weeks, serum levels of total proteins, albumin, total cholesterol, HDL cholesterol and triglycerides were evaluated using commercial kits (Labtest Diagnóstica S.A., Brazil) after 16 hours fasting. Liver lipid quantification was analyzed according to the Folch method [50]. Briefly, frozen liver tissue (100mg) was homogenized in 950μL of chloroform:methanol (2:1). 200μL of methanol were added to the mixture and the samples were centrifuged. The supernatant was transferred to a weighted clean tube, then mixed with 400μL of saturated saline solution. After centrifugation of the mixture, the upper phase was discarded and the lower chloroform phase containing the lipids was washed three times with a Folch solution (2% NaCl 0.2%, 3% Chloroform, 47% distillated water, 48% methanol), evaporated under 60°C temperature, and total lipid weight was determined.

### Real-time PCR

Epididymal white adipose tissue was collected for the analysis of gene expression using qRT-PCR. Total RNA from the samples was extracted using TRizol reagent (Sigma-Aldrich, USA) according to the manufacture’s protocol. Reverse transcription was performed to obtain cDNA. mRNA amplification was determined using SYBR Green reagent (Solis BioDyne, Estonia). Gene expression was normalized to endogenous glyceraldehyde-3-phospate dehydrogenase (GAPDH), and the results are expressed as arbitrary units. The primers were–GAPDH: 5’-CTCAAGATTGTCAGCAATGC-3’ and 5’-CAGGATGCCCTTTAGGTGGGC-3’; HSL: 5’ACCGAGACAGGCCTCAGTGTG-3’ and 5’-GAATCGGCCACCGGTAAAGAG-3’; Leptin: 5’-CCTGTGGCTTTGGTCCTATCTG-3’ and 5’-AGGCAAGCTGGTGAGGATCTG-3’; PPAR γ: 5’-ACAGACAAGATTTGAAAGAAGCGGTGA-3’ and 5’-TCCGAAGTTGGTGGGCCAGA-3’; UCP1: 5’-GTGAACCCGACAACTTCCGAA-3’ and 5’-TGCCAGGCAAGCTGAAACTC-3’.

### Isolation of the adipose tissue stromal vascular fraction

For isolation of the stromal vascular fraction (SVF) from adipose tissue [51], epididymal white adipose tissue (EWAT) was collected and then minced in DMEM, containing 4% Bovine Albumin Serum Fatty Acid Free and 0.1% glucose. Collagenase VIII (Sigma-Aldrich, Merck KGaA, USA) was added, at 4 mg/g of tissue, to the mixture containing the minced tissue followed by incubation at 37°C under constant agitation for 40 minutes. Following centrifugation, floating adipocytes were separated and the SVF pellet ressuspended and analyzed.

### Cell culture

Adipocytes (3x10^5^ cells/mL) and cells from SVF (5x10^6^ cells/mL) were cultured in 5% CO_2_ incubator at 37°C for 24 hours in the absence or presence of PMA (0.4mg/mL) and Ionomycin (5mg/mL). After incubation, the supernatant was collected and stored at -70°C until use. Cytokine levels were assessed by Cytometric Bead Array (CBA) Mouse Th1/Th2/Th17 (BD Biosciences, USA) and adiponectin secreted by the culture of adipocytes was assessed by ELISA (R&D System, USA).

### Flow cytometry

For analysis of eosinophils, SVF cells were stained with antibodies against CD11b (M1/70; BioLegend, USA), and Siglec F (E50-2440; BD Biosciences, USA) flowed by fixation with paraformaldehyde. Data from cell acquisition were analyzed and after defining the intersection population from the gates of leucocytes, single cells and time, cells stained with CD11b^int^SiglecF^+^ were considered eosinophils (S1 Fig).

For analysis of lymphocyte and innate lymphocyte subsets, SVF cells were stained with antibodies against CD3 (17A2; BioLegend), CD4 (GK1.5; BioLegend), CD45 (30-F11; BioLegend), CD 127 (SB/199; BioLegend) and Lin^-^ (CD11b –M1/70, BioLegend; CD11c –HL3, BioLegend; CD16–93, BioLegend; CD19 – 1D3, BioLegend; FcεRIa–MAR1, BioLegend) then fixed and permeabilized with fix/perm buffer (eBioscience, Thermo Fisher Scientific, USA) according to the manufacturer’s instructions. Cells were then incubated with antibodies against Tbet (4B10; BioLegend), ROR γT (Q31-378; BD Biosciences) and Gata3 (16E10A23; BioLegend). Lymphocytes were first identified by forward/side scatter dot plot, then doublets and possible interruptions in the acquisition were excluded. Positive cells for CD3 and CD4 were selected. Then CD3^+^CD4^+^Tbet^+^ were considered Th1 cells, CD3^+^CD4^+^Gata3^+^ Th2 and CD3^+^CD4^+^RORγT^+^ Th17 (S2 Fig). To identify ILC2 cells, after sequentially gating lymphocytes, single cells and time, Lin^-^ population was selected by gating Lin^-^ x CD3. Then we identify CD45^+^CD127^+^Gata3^+^ as ILC2 (S3 Fig).

For characterization of Treg subtypes, SVF cells were stained with antibodies against CD3 (17A2; BioLegend), CD4 (GK1.5; BioLegend), GITR (YGITR 765; BioLegend), LAP (TW7-16B4; BioLegend), CD25 (PC61; BioLegend), CD134 (OX-86; BioLegend) and CD152 (UC10-4B9; BioLegend), then fixed and permeabilized with fix/perm buffer. Cells were then incubated with antibody against Foxp3 (150D; BioLegend). CD3^+^CD4^+^ T lymphocytes were selected as described above and then population positive for CD25 and Foxp3 was selected. CD3^+^CD4^+^CD25^+^Foxp3^+^ cells were considered Tregs and analyzed for surface marker (S4 Fig).

Flow cytometry data were collected on BD LSRFortessa Flow Cytometer using BD FACSDiva Software and gates were set according to unstained cells using FlowJo (version 10.5.3, Tree Star Inc, USA).

### *In vitro* Treg suppression assays

Tregs from EWAT were isolated from SVF cells using Dynabeads FlowComp Mouse CD4^+^CD25^+^ Treg Cells Kit (Invitrogen, Thermo Fisher Scientific, USA). T effector cells (Teff), isolated from the spleen of a control animal (non-infected and fed with regular chow) using the same kit, were stained with 5μM CFSE (Invitrogen, USA) for 10 minutes. Functional Treg assay was performed as described [52]. Briefly, Teff cells were co-cultured with Tregs at indicated proportions and stimulated with Dynabeads Mouse T-Activator CD3/CD28 (Life Technologies, USA) for 72 hours in 5% CO_2_ incubator at 37°C. Cells were acquired in BD LSRFortessa Flow Cytometer and the decay in CFSE fluorescence was analyzed with FlowJo (version 10.5.3, Tree Star Inc) using the tool Proliferation Modeling.

### Statistical analysis

All results were expressed as mean ± standard error of the mean. Group means were compared by Mixed-effects analysis or two-tailed Student’s test using GraphPad Prism (version 9.0.0, GraphPad Software Inc, USA). Probability values below 0.05 were considered statistically significant.

## Results

### *H*. *polygyrus* infection attenuated weight gain and metabolic dysfunctions

**Fig 1:**
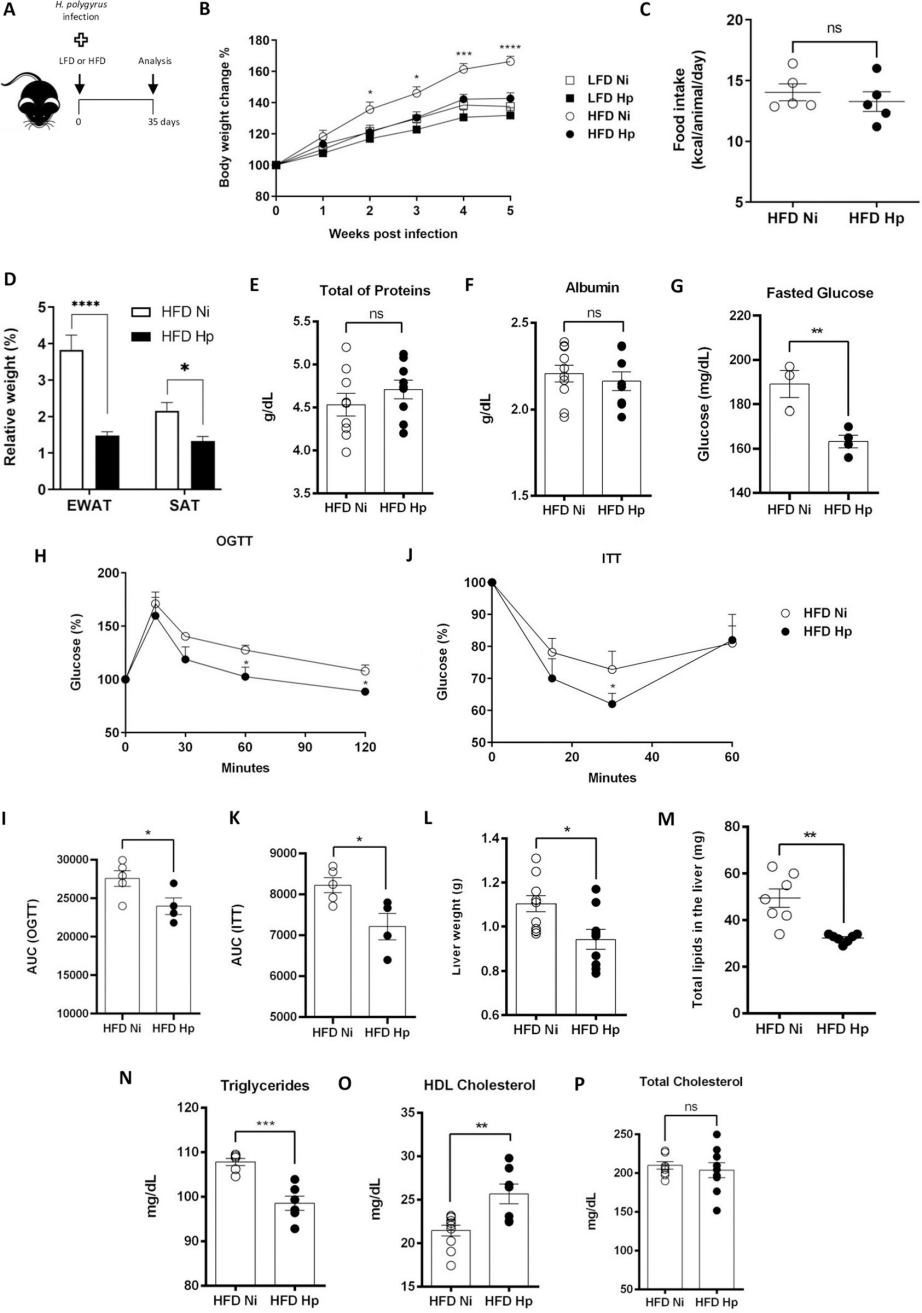
*H*. *polygyrus* infection attenuated weight gain and prevented metabolic dysfunctions in early stages of HFD-induced obesity. Animals were treated with control low fat diet (LFD) or with obesogenic high fat diet (HFD) and infected with *H*. *polygyrus* (Hp) or left uninfected (Ni) for five weeks (A). Body weight (B), caloric intake (C), relative weight of epididymal white adipose tissue (EWAT), and subcutaneous adipose tissue (SAT) (D) were assessed. Serum levels of total proteins (E) and albumin (F) were evaluated at the end of experiment. Fasting glycemic levels (G), response to oral glucose tolerance tests after glucose gavage (OGTT) (H and I) and insulin tolerance tests (ITT) after intraperitoneal injection of insulin (J and K) were compared between HFD-fed groups. Liver mass (L), hepatic quantification of lipids (M), fasting levels of serum triglycerides (N), HDL cholesterol (O) and total cholesterol (P) were assessed. N = 4–10 pooled from two or more experiments performed. Two-tailed T-Test was used to compare HFD Hp and HFD Ni groups. * p<0.05 ** p<0.01 *** p<0.001 **** p<0.0001 between HFD groups. Images (A) were used from Wikimedia Commons (https://commons.wikimedia.org).

To determine the effect of *H*. *polygyrus* infection on early stages of obesity induced by high fat diet (HFD), C57BL/6 male mice were infected or not with L3 *H*. *polygyrus* larvae and fed with HFD diet for 5 weeks (Fig 1A). The infection was able to prevent exacerbated weight gain in animals fed with HFD (Fig 1B) without decreasing the amount of food intake (Fig 1C). The lower weight gain in the HFD Hp group was associated with decreased weights of epididymal (EWAT) and subcutaneous (SAT) adipose tissue (Fig 1D). Importantly, although the weight gain curve of the infected animals receiving control diet (low fat diet, LFD) seems somewhat reduced when compared to the non-infected group, this difference was not statistically significant (Fig 1B), which suggests that the differences between infected and non-infected groups treated with HFD are not due to parasite spoliation of the host. Another data that suggests the host is not being spoliated by the parasite is that neither total serum protein or serum albumin levels differed between infected and non-infected animals (Fig 1E and 1F). Therefore, we conclude that *H*. *polygyrus* infection improved weight control in animals under HFD without causing malnutrition.

Obesity is associated with dysregulation of glucose metabolism and hepatic steatosis [12,27]. Animals fed with HFD and infected with *H*. *polygyrus* presented lower fasting glycemic levels when compared to non-infected controls (Fig 1G). Further, HFD Hp group had a better response to glucose tolerance test showed by the faster return to blood basal levels after glucose injection, when compared to non-infected controls (Fig 1H and 1I). Both results indicate that the presence of the infection improved the development of glucose metabolic dysfunction associated to weight gain. Insulin sensitivity was also improved in infected animals when compared to the non-infected group. We found that blood glucose levels, after insulin injection, were higher in HFD Ni mice when compared to HFD Hp group, which suggested that the infection prevented the development of peripheral insulin resistance (Fig 1J and 1K). In addition, infected animals under HFD had significantly lower liver mass when compared to HFD Ni group (Fig 1L). This decrease in liver mass was directly associated with the amount of lipid/gram of liver, which was also lower in infected animals (Fig 1M). Furthermore, the infection also improved dyslipidemia associated with the HFD, i.e., decreased serum levels of triglycerides (Fig 1N) and also increased levels of HDL cholesterol (Fig 1O), besides not changing total cholesterol (Fig 1P). Overall, the effects of *H*. *polygyrus* infection on experimental obesity is in agreement with other models described in literature that helminth infection improves weight gain, fat accumulation and metabolic syndrome in animals fed with HFD [35–41].

### *H*. *polygyrus* infection prevented the establishment of inflammation caused by high fat diet

**Fig 2:**
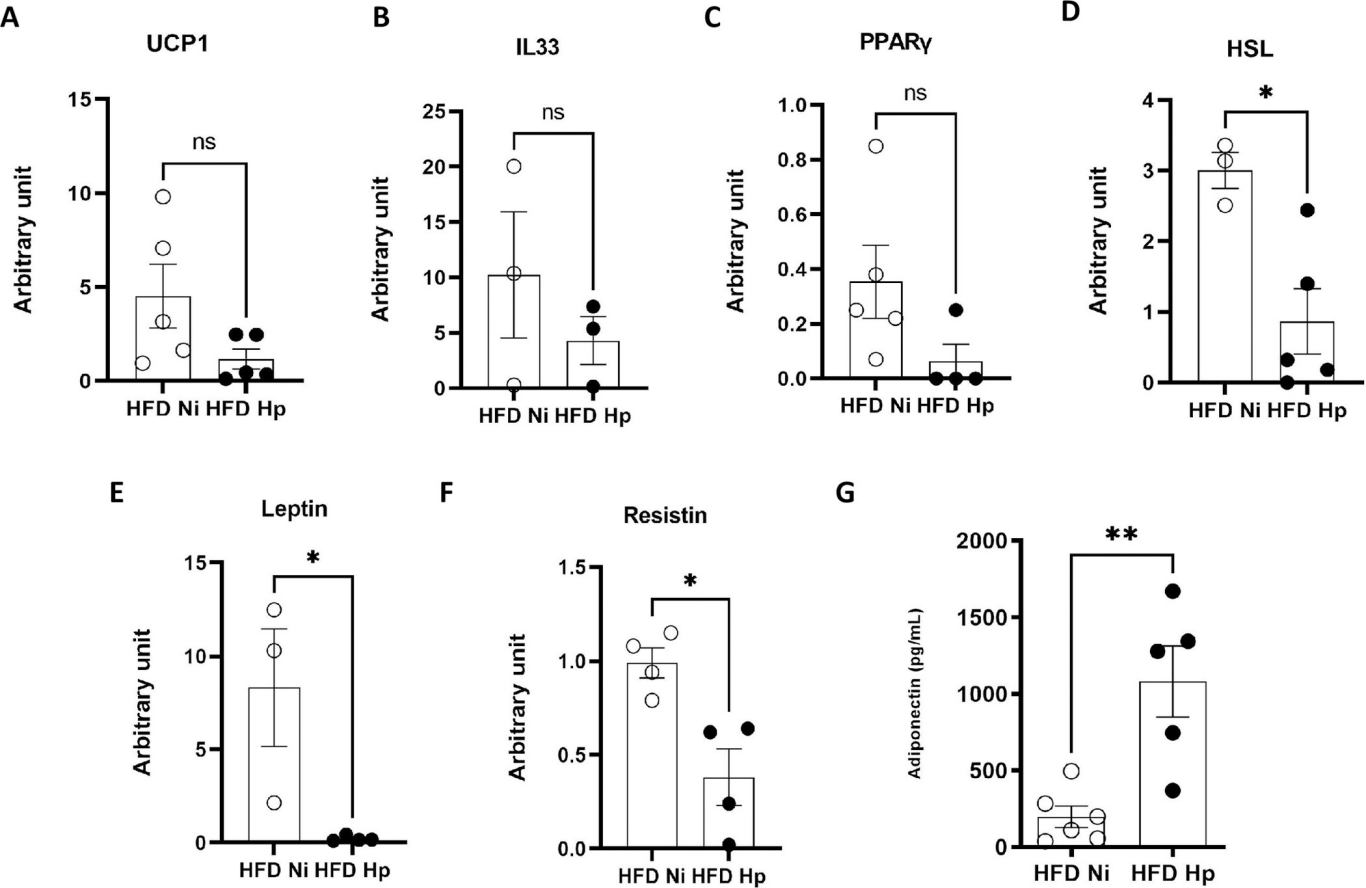
*H*. *polygyrus* infection prevented the dysregulation in the production of adipokines due to high fat diet consumption. Expression of UCP1 (A), IL33 (B), PPARγ (C), hormone-sensitive lipase (HSL) (D), Leptin (E) and Resistin (F) by EWAT cells, measured by qPCR. Production of Adiponectin by cultured adipocytes, measured by ELISA (G). n = 3–7 pooled from two experiments performed. Two-tailed t test. * p<0.05 ** p<0.01 between HFD groups.

In order to search possible mechanisms induced by *H*. *polygyrus* that could improve the metabolic parameters, we collected EWAT and measured the expression of some transcriptional factors and hormones described to play a role in the metabolism. Uncoupling proteins (UCP’s) are known to catabolize stored energy to generate heat, which ends up diminishing the effects of metabolic diseases, like obesity and type II diabetes [53]. Also, the expression of UCP1 is linked to ILC2 frequency, which is induced by IL33. PPAR’s (Peroxisome proliferator-activated receptors), specially PPARγ, are responsible for regulating fatty acid storage and glucose metabolism by increasing insulin sensitivity [54]. Infection with Hp did not modulate the levels of mRNA for UCP1 (Fig 2A), IL33 (Fig 2B) or PPARγ (Fig 2C) in EWAT of HFD-treated animals at 5 weeks post infection suggesting a minor role of these factors in Hp-associated weight control.

In contrast, HFD Hp group displayed lower expression of hormone-sensitive lipase (HSL) (Fig 2D) suggesting improved sensitivity to insulin [55]. We could also observe reduced expression of leptin (Fig 2E), resistin (Fig 2F), and higher production of adiponectin (Fig 2G) by EWAT cells from HFD Hp mice, which are consonant findings with metabolic improvements [56–59].

**Fig 3:**
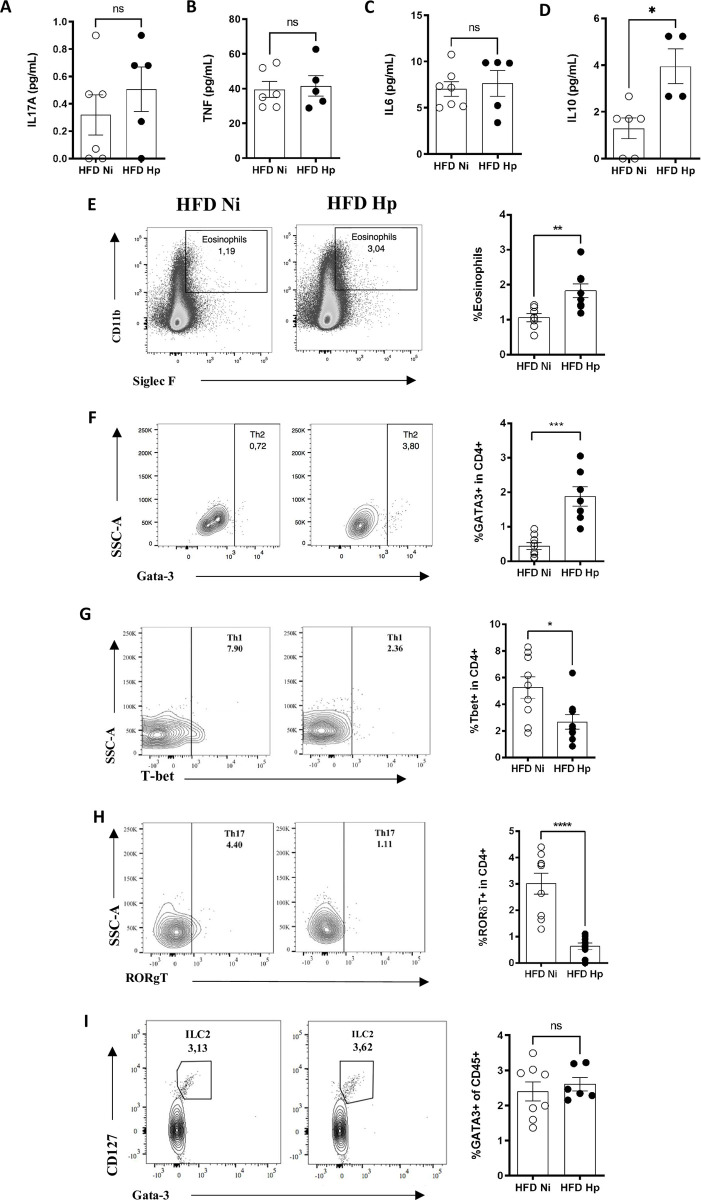
*H*. *polygyrus* infection prevented the establishment of inflammation caused by high fat diet. Production of IL17A (A), TNF (B), IL6 (C) and IL10 (D) by stromal vascular fraction cells from epididymal white adipose tissue (EWAT). Representative gates and frequency of eosinophils (E), Th2 (F), Th1 (G), Th17 (H) and ILC2 (I) cells isolated from EWAT and analyzed by flow cytometry. n = 7–10 pooled from two or more experiments performed. Two-tailed t test. * p<0.05 ** p<0.01 *** p<0.001 **** p<0.0001 between HFD groups.

Adipose tissues also secrete cytokines that are associated with the development of metabolic disorders, known as adipokines [12]. Due to the importance such cytokines in the metabolism, we analyzed their production by EWAT. SVF cells were separated from adipocytes, cultured for 24 hours, and then cytokines were measured in the supernatants. We found no difference in the secretion of inflammatory cytokines like IL17A (Fig 3A), TNF (Fig 3B) and IL6 (Fig 3C) by SVF cells when comparing HFD Hp and HFD Ni groups. Production of IL2, IL4 and IFNγ were not detected in the culture of SVF cells. On the other hand, the production of homeostatic adipocytokines like IL10, by SVF cells (Fig 3D), was increased in HFD Hp group when compared to HFD Ni. These data suggest that the effect of *H*. *polygyrus* infection in metabolic parameters might be related to the secretion of anti-inflammatory or homeostatic adipocytokines, rather than a reduction of pro-inflammatory cytokines.

To gain further insights about how the infection was modulating the inflammatory profile in adipose tissue, we evaluated the phenotype of the resident immune cells. We observed that the infection by *H*. *polygyrus* was associated with increased percentage of eosinophils (Fig 3E) and Th2 cells (Fig 3F) in the epididymal white adipose tissue. On the other hand, Th1 (Fig 3G) and Th17 (Fig 3H) cells were downmodulated by infection. ILC2 frequencies (Fig 3I) did not differ between both groups. Together, our data showed that the presence of the Hp infection is associated with an increased Th2 cells and decreased Th1 and type 17 infiltration in adipose tissue, despite maintenance of IL17A, TNF and IL6, that may have innate immune cells sources.

### *H*. *polygyrus* infection improved the number, phenotype and function of Tregs in adipose tissue

**Fig 4:**
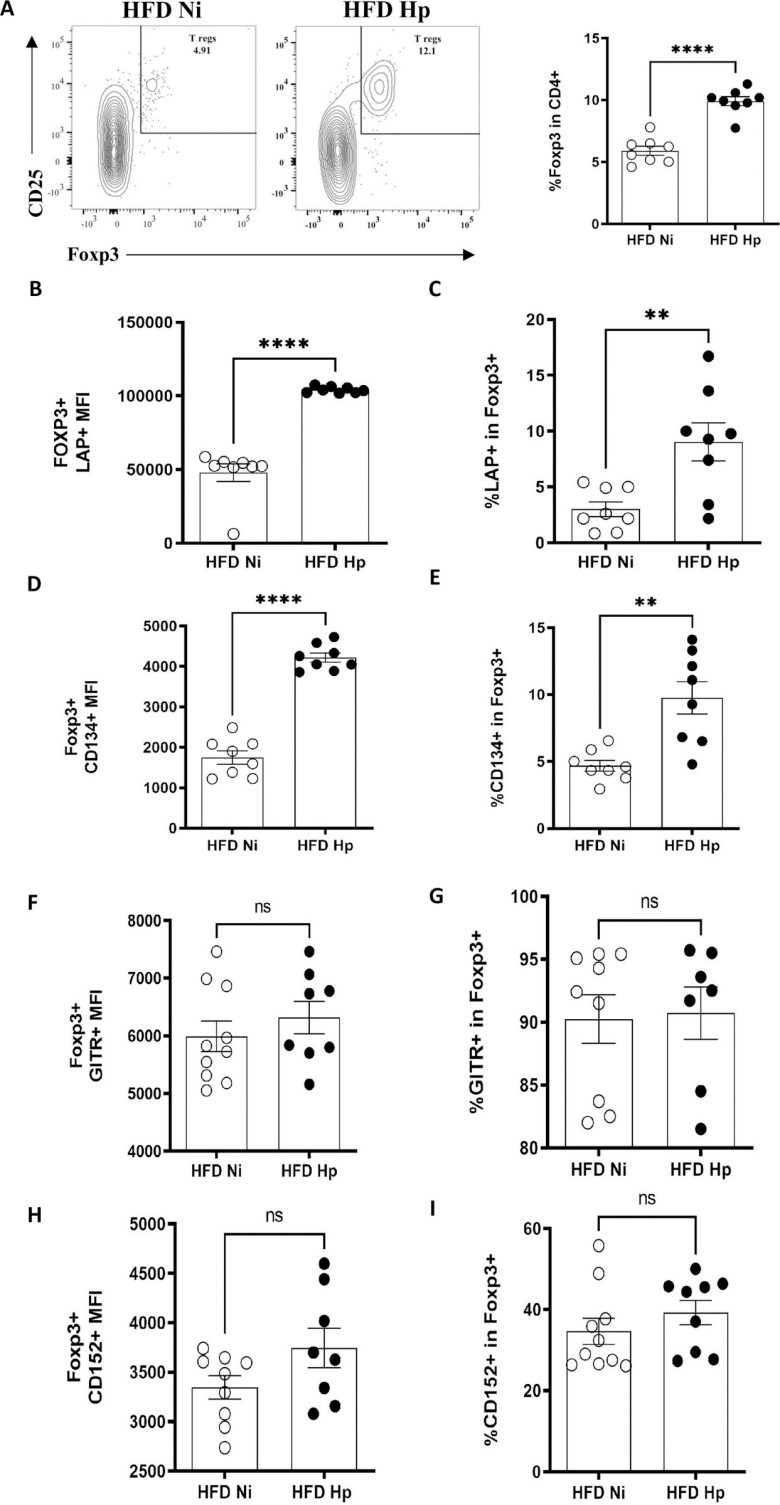
*H*. *polygyrus* infection induced alterations in number and phenotype of Tregs from EWAT. Representative Contour Plots and percentage of Tregs (A) in HFD Ni and HFD Hp groups, analyzed by flow cytometry. Tregs gated on CD3^+^CD4^+^CD25^+^Foxp3^+^. Mean Fluorescence Intensity (MFI) (B) and percentage (C) of LAP in Foxp3^+^ cells. MFI (D) and percentage (E) of CD134 in Foxp3^+^ cells. MFI (F) and percentage (G) of GITR in Foxp3^+^ cells. MFI (H) and percentage (I) of CD152 in Foxp3^+^ cells. n = 10 pooled from two experiments performed. Two-tailed t test. ** p<0.01 ****p<0.0001 between HFD groups.

Obesity is associated not only with increased pro-inflammatory background, but also with Treg dysfunction [47,60]. On the other hand, despite the fact that helminth infections have been associated with improved Treg activity [25,29,33,34,61], their phenotype in obesity-helminth infection interface has been poorly investigated. To gain insights about how helminth infection can influence the biology of Tregs in obesity, we analyzed the abundance of Tregs in adipose tissue and also the expression of cell surface markers in Tregs [28]. *H*. *polygyrus* infection increased the frequency of Tregs in adipose tissue (Fig 4A) in HFD-treated animals and modulated their phenotype. For example, the expression of LAP, a marker of membrane-bound TGFß [62], was increased in Tregs of Hp-infected animals when compared to HFD Ni group at MFI (Fig 4B) and frequency levels (Fig 4C). The infection also increased the frequency and per cell expression of CD134 by adipose tissue Tregs (Fig 4D and 4E). On the other hand, the expression of GITR (Fig 4F and 4G) and CTLA4/CD152 (Fig 4H and 4I) by adipose tissue Tregs were not altered by the infection.

**Fig 5:**
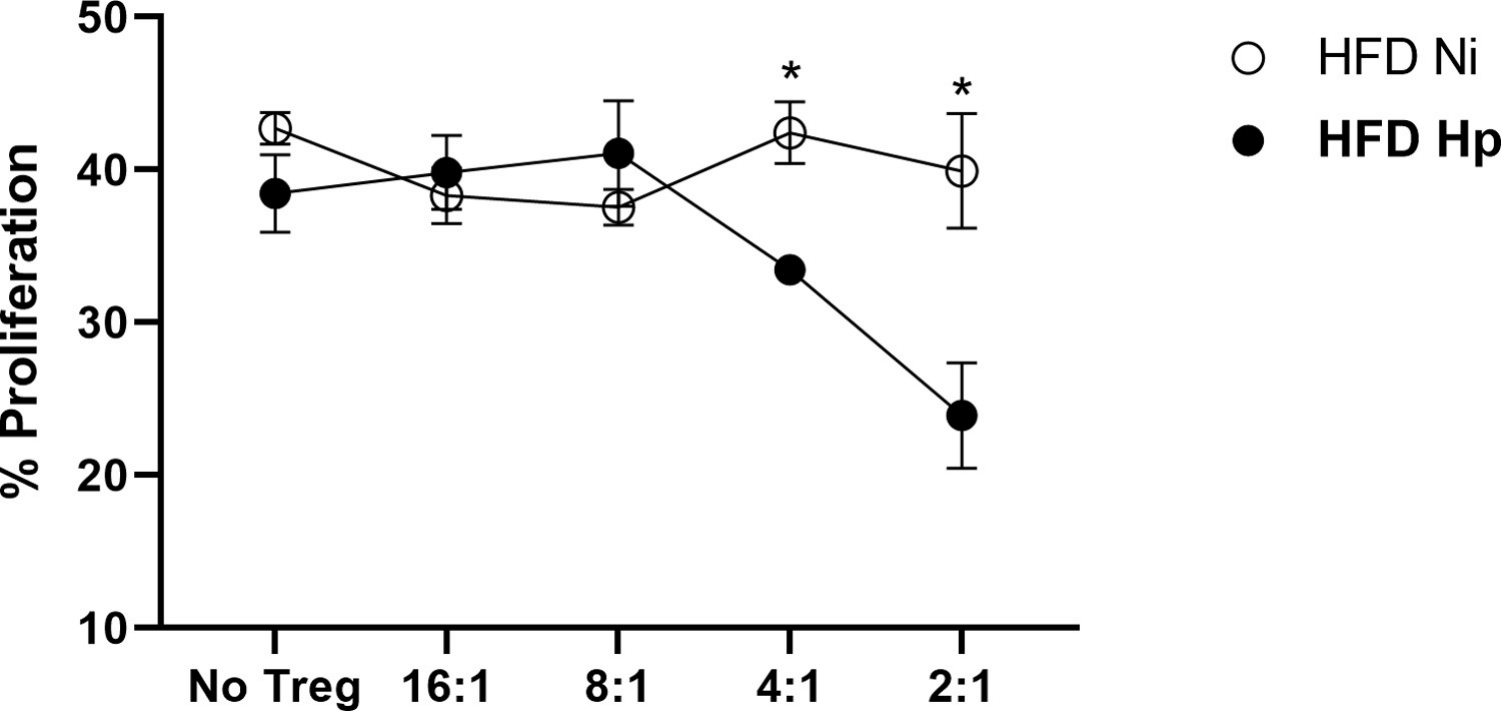
*H*. *polygyrus* infection prevented loss of function by adipose tissue Tregs. Proliferation percentage, measured by CFSE decay, of non-Tregs cells after co-culture with different concentrations of Tregs. n = 10 pooled from two experiments performed. Two-tailed t test. * p<0.05 between HFD groups.

Since the infection altered the expression of functional markers of Tregs, we verified if it could also impact the Treg function in the context of obesity. Adipose tissue Tregs were isolated and used in a proliferation inhibition assay with splenic non-Tregs T cells from LFD-treated animals. Animals from HFD Ni group showed an important dysfunction in Tregs since they were unable to inhibit T cell proliferation at any concentration tested (Fig 5). On the other hand, *H*. *polygyrus* infection was able to revert this diet-associated dysfunction. Tregs from adipose tissue of infected animals were able to inhibit T cell proliferation at the proportions of 4:1 and 2:1. Together our data showed that *H*. *polygyrus* infection can modulate different aspects of Treg cells resident in adipose tissue.

## Discussion

Studies on the effects of helminth infections in metabolic diseases are still emerging and are still controversial, especially regarding the immunological mechanisms at play in the improvement of metabolic parameters in the host. Our data corroborate previous publications that helminth infections are able to prevent HFD-induced exacerbated weight gain [35,39,40], dysfunctional glucose and lipid metabolism [37,41], and decrease obesity-associated inflammation [35,36,38,40]. Previous reports have associated these effects with a variety of mechanisms such as increased adipose tissue content of eosinophils [37,40,63], ILC2 infiltration [37], M2 polarization [35,36,40], PPARγ activation [35,64], increased expression of IL33 [65], and microbiota modulation [66] indicating that helminth infection might employ a variety of mechanisms to influence metabolic syndrome, especially inducing type 2 immune response polarization which should counter regulate the obesity-associated inflammation with high levels of IL6 [25], IFNγ [25], TNF [67], IL1ß [68] and CCL2 [69]. Interestingly, obesity is not only considered an inflammatory disease, but the inflammation itself has been described as necessary for weight gain and development of metabolic dysregulation [27,70].

In obese humans, the presence of an active helminth infection is associated with lower levels of LDL cholesterol [71] and improved glucose metabolism with decreased levels of fasting glucose and prevention of development of metabolic syndrome and type 2 diabetes [72], even if infection did not provide major impact on Body Mass Index [73] and HOMA-IR [71]. As in animal models, helminth-associated metabolic improvement in humans have been linked to increased Th2 and decreased Th1 responses [73], and modulation of the secretion of chemokines [74], cytokines [74] and adipokines [73].

Indeed, we observed decreased frequencies of Th1 and Th17, and increased Th2 cell infiltration in adipose tissue, which is consistent with the mechanisms speculated to be at play in helminth-associated improvement of obesity parameters i.e., decrease in weight gain and amelioration of metabolic syndrome. ILC2 did not seem to have a role in our model, since there was no difference in the frequency of these cells between infected and non-infected groups. The increase of ILC2 is dependent on the secretion of IL33, and the elevated prevalence of this cells is described to induce the expression of UCP1 [65]. In our experiments, IL33 expression did not differ between HFD Ni and HFD Hp groups and we also saw no difference in either ILC2 or UCP1, suggesting that these mechanisms might be playing a minor role the metabolic improvements we observed, in contrast to previously described data [65]. Interestingly, SVF cells isolated from HFD Hp animals secreted similar levels of IL17, TNF and IL6 when compared to HFD Ni animals, which might be related to differences associated with ex vivo and in vitro experimentation, or to additional mechanisms beyond inflammation regulation. On the other hand, adipokines were modulated by infection. Adipokines are key substances produced by adipose tissue cells that exert impact not only in the tissue, but in the overall metabolism [56]. During the process of weight gain, the production of adipokines shifts adding to metabolic disturbances [57]. For example, increased levels of leptin and resistin are associated with elevated caloric intake [75] and insulin resistance [76,77]. In contrast, adiponectin stimulates insulin function [12,58,59] and sensitivity [59], and its levels are negatively correlated with waist circumference, visceral fat weight, triglycerides and HDL levels, fast glucose and insulin levels, and also with the development of type 2 diabetes [78–80]. Interestingly, leptin and adiponectin play opposite roles to Treg balance: while leptin inhibits Treg function [81,82], adiponectin promotes regulatory functions, including IL10 production [83]. IL10 possess various cellular sources, being Tregs a major producer. Although recent studies have suggested that IL10 have deleterious effect on insulin pathways and weight gain during experimental obesity [84,85], when it comes to the context of helminth infection and high fat diet, increased secretion of IL10 is known to control the development of type 2 diabetes [38], improve triglycerides levels [36], and increase sensitivity to insulin [41]. Perhaps, the association between IL10 and type 2 immunity observed in helminth infections, the modified Th2, may shift the impact of IL10 on obesity. In addition, IL10 has also been shown to prevent TNF-induced fat accumulation in the liver [86]. Indeed, we could observe improvement in triglycerides levels, insulin sensitivity, and fat accumulation in the liver in HFD Hp animals that displayed higher production of IL10.

Tregs from visceral adipose tissue are associated with insulin sensitivity and maintenance of metabolic homeostasis, comprising about 50% of CD4^+^ cells in this tissue [87]. The development of obesity has been linked to a shift from regulatory to pro-inflammatory environment in adipose tissue [27]. Studies have found that obesity is associated with reduced number [28] and dysfunction of Tregs [47]. Tregs from visceral adipose tissues display unique features like expression of leptin and adiponectin receptors being able to respond to adipokines [81] and are regulated by PPARγ [64], presenting characteristic gene expression. They are also highly responsive to IL33 [88], although we did not find a role for IL33 in our model. Surprisingly, most studies on helminth and obesity have focused on the role of type 2 immune responses, while little is known about Treg cells in the helminth-obesity interaction. We observed that helminth infection was able to modulate the phenotype, and to improve frequency and function of Tregs in the adipose tissue of mice fed HFD. Due to its nature and function, we speculate that Tregs can be directly associated with the regulation of inflammatory parameters like the higher secretion of IL10, and metabolic improvements [28,89]. Interestingly, the expression of LAP, one of the receptors found to be increased by helminth infection, in Tregs cells is already described to improve glycemic levels, decrease the secretion of inflammatory mediators, impair accumulation of liver fat and reduce hyperplasia in ß-pancreatic cells [47,90]. CD134, another receptor upregulated by the infection in Tregs, is known to reduce Th1 and Th17 cells differentiation and to sustain Tregs suppression function [91–93]. Taken together, these data suggest that Tregs may be implicated in the mechanisms induced by helminth infection in regulation of obesity different than those associated with type 2 polarization.

Our data are one of the firsts to imply Treg in helminth-associated modulation of obesity and metabolic syndrome. In addition, helminths have been shown to modulate Tregs in circulation [94], lymph nodes [33,34] and spleen [32,36], but this is the first time showing that helminths also modulate adipose Tregs. Additional experiments are still necessary to determine the exact role of Tregs in helminth’s modulation of obesity, like the depletion of Tregs. Nonetheless, the depletion of Tregs is known to spontaneously exacerbate inflammation, inducing immune activation, e.g., increasing the secretion of IFN and Th17 differentiation [95]. In the context of diabetes, the ablation of Tregs resulted in rapid increase of natural killer cells, that induced insulitic lesions, resulting in diabetes after three days of depletion [96]. Increased production of IL12 by dendritic cells and changed gene-expression program of local CD4^+^ cells were also observed [96]. In addition, it is not possible yet to determine if control of weight gain and improvement of metabolic syndrome is a consequence of Treg function improvement by helminths. However, since Tregs are sensitive to and also modulators of adipokines and cytokines with implications in obesity.

Taken together, our data show the beneficial effects of helminth infection in early stages of HFD-induced obesity and its associated metabolic dysfunctions, implicating Tregs in the helminth-obesity interface. These effects can be attributed to several interrelated or independent events resulting from *H*. *polygyrus* infection: e.g., increased secretion of IL10 and adiponectin, increased eosinophils frequency, promotion of Th2 differentiation, increased frequency of Tregs, increased expression of Tregs markers like LAP and CD134 and maintenance of Tregs functionality. Understanding the influence of helminth infection on regulatory mechanisms that may alleviate metabolic syndrome may bring novel approaches to treat or prevent obesity.

## Supporting information

S1 Fig Gate strategy analysis of eosinophils. Dot/contour plots are representative of the analysis strategy used. Initially gates for leucocytes (SSC-A x FSC-A), single cells (FSC-H x FSC-A) and time (SSC-A x Time) were delimited. Then after mixing the gates using the tool Boolean Gate–Make and Gate, the eosinophils population was determined by CD11b^int^Siglec F^+^.
(TIF)

S2 Fig Gate strategy analysis for T lymphocytes subtypes. Dot/contour plots are representative of the analysis strategy used. After selecting the lymphocytes population (SSC-A x FSC-A), the gate of single cells (FSC-H x FSC-A) was delimited considering only the cells included in the previous gate. If necessary, considering the population from single cells, the gate of time was made (SSC-A x Time) to exclude interruptions during acquisition. CD3^+^ cells flowed by CD4^+^ were identified by being T helper cells. This last population was analyzed considering SSC-A x Tbet/Gata3/RORγT, resulting in Th1, Th2 and Th17 populations, respectively.
(TIF)

S3 Fig Gate strategy analysis for ILC2. Dot/contour plots are representative of the analysis strategy used. After selecting the lymphocytes population (SSC-A x FSC-A), the gate of single cells (FSC-H x FSC-A) was delimited considering only the cells included in the previous gate. If necessary, considering the population from single cells, the gate of time was made (SSC-A x *Time*) to exclude interruptions during acquisition. Lin^-^ cells flowed by CD45^+^ were identified. This last population was analyzed considering CD127 x Gata3, resulting in a double positive population, identified as ILC2.
(TIF)

S4 Fig Gate strategy analysis of Tregs. Dot/contour plots are representative of the analysis strategy used. First the gate of lymphocytes (SSC-A x FSC-A) was delimited, and from it the gate for single cells (FSC-H x FSC-A) was made. From the resulted population CD3^+^ followed by CD4^+^, and CD25^+^ x Foxp3^+^ cells were selected resulting in Tregs. Considering Tregs the gates SSC-A x GITR, CD152, LAP and CD134 were made resulting in the positive population for each marker.
(TIF)
