# Supplementary figures and images for: Helminth infection modulates number and function of adipose tissue Tregs in high fat diet-induced obesity
Source: PLoS Negl Trop Dis. 2022 May 2;16(5):e0010105. doi: 10.1371/journal.pntd.0010105 (PMC9098094; doi:10.1371/journal.pntd.0010105)

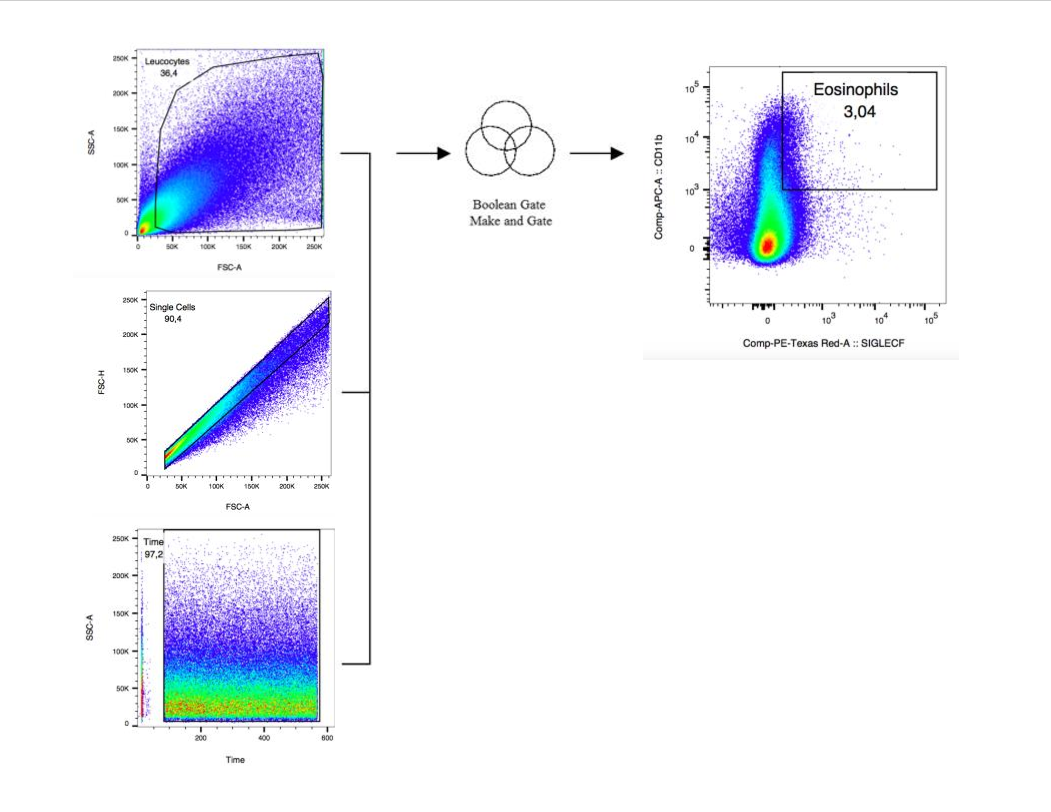

Supplement: S1 Fig — Dot/contour plots are representative of the analysis strategy used. Initially gates for leucocytes (SSC-A x FSC-A), single cells (FSC-H x FSC-A) and time (SSC-A x Time) were delimited. Then after mixing the gates using the tool Boolean Gate–Make and Gate, the eosinophils population was determined by CD11bintSiglec F+. (TIF) [file pntd.0010105.s001.tif]

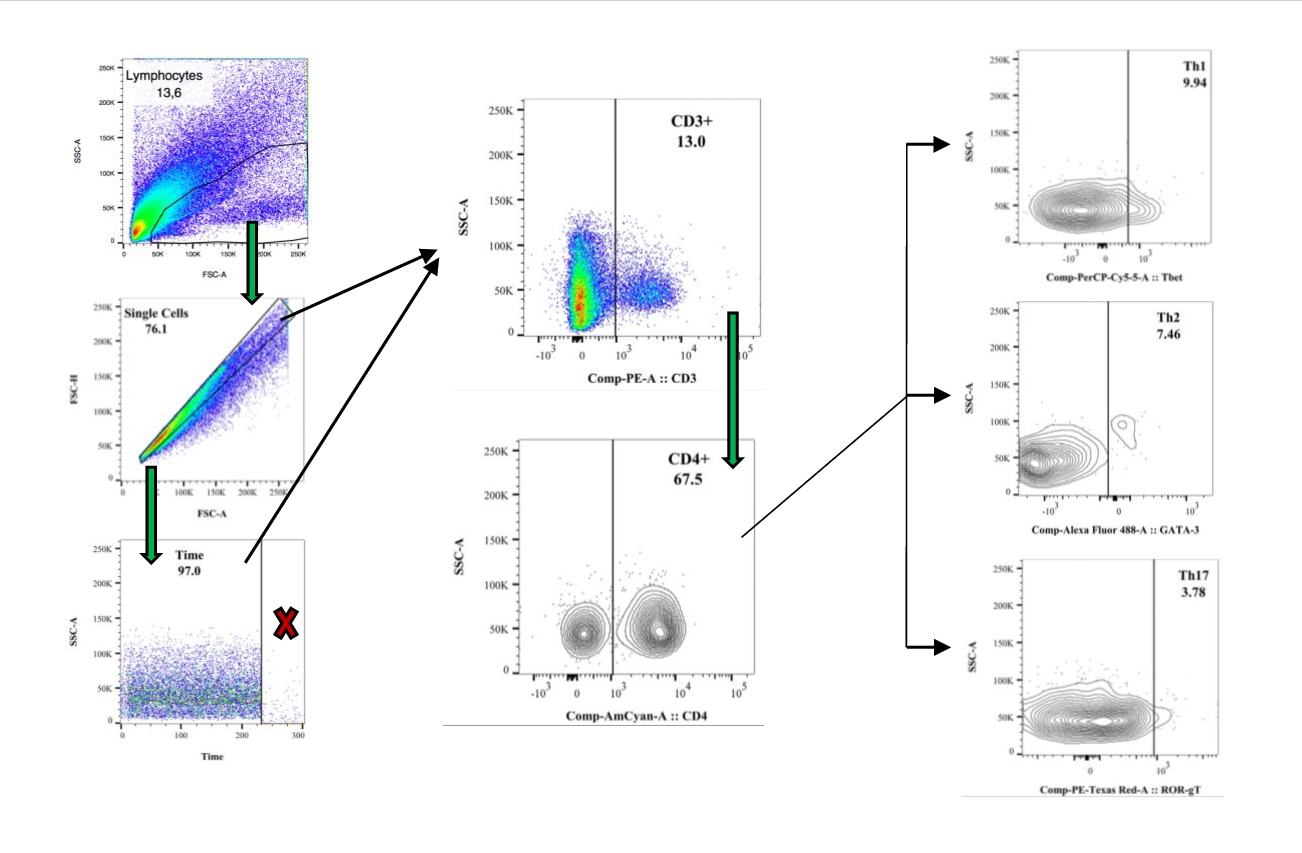

Supplement: S2 Fig — Dot/contour plots are representative of the analysis strategy used. After selecting the lymphocytes population (SSC-A x FSC-A), the gate of single cells (FSC-H x FSC-A) was delimited considering only the cells included in the previous gate. If necessary, considering the population from single cells, the gate of time was made (SSC-A x Time) to exclude interruptions during acquisition. CD3+ cells flowed by CD4+ were identified by being T helper cells. This last population was analyzed considering SSC-A x Tbet/Gata3/RORγT, resulting in Th1, Th2 and Th17 populations, respectively. (TIF) [file pntd.0010105.s002.tif]

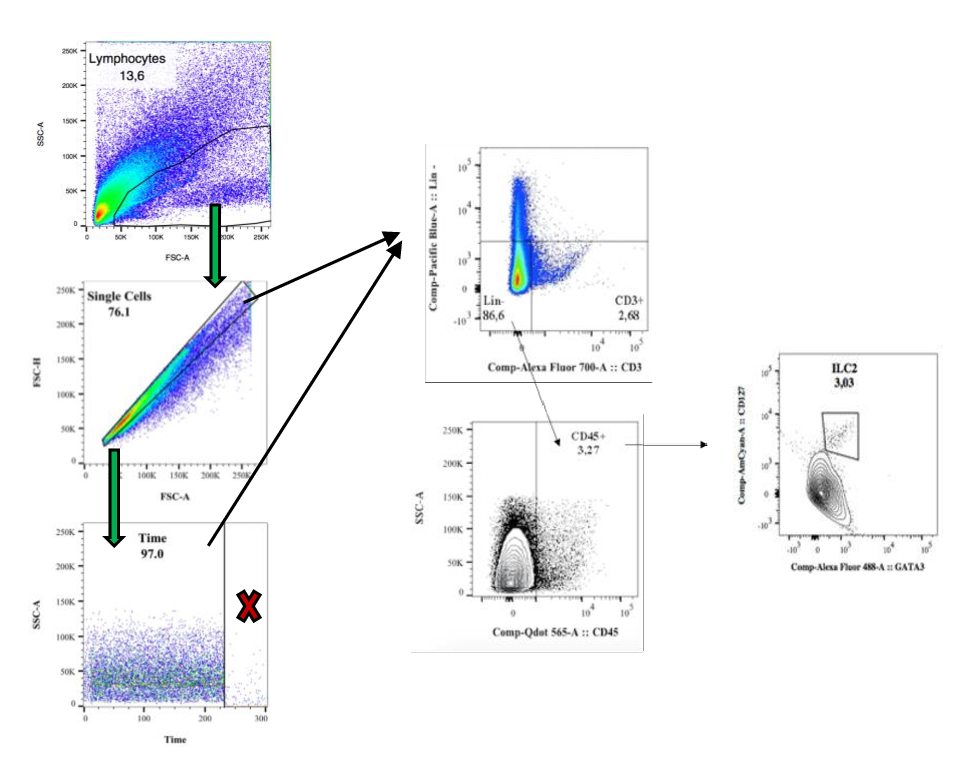

Supplement: S3 Fig — Dot/contour plots are representative of the analysis strategy used. After selecting the lymphocytes population (SSC-A x FSC-A), the gate of single cells (FSC-H x FSC-A) was delimited considering only the cells included in the previous gate. If necessary, considering the population from single cells, the gate of time was made (SSC-A x Time) to exclude interruptions during acquisition. Lin- cells flowed by CD45+ were identified. This last population was analyzed considering CD127 x Gata3, resulting in a double positive population, identified as ILC2. (TIF) [file pntd.0010105.s003.tif]

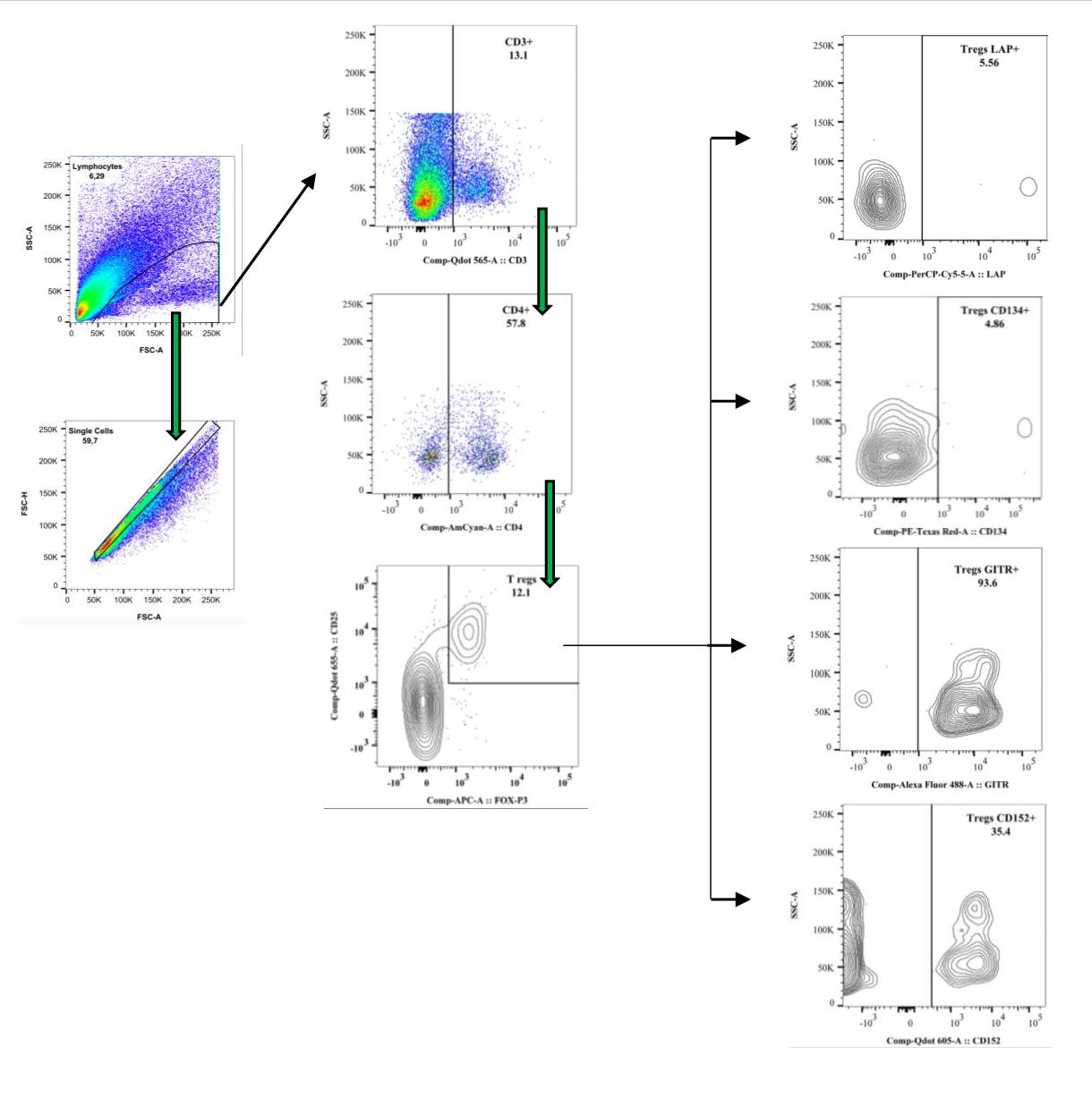

Supplement: S4 Fig — Dot/contour plots are representative of the analysis strategy used. First the gate of lymphocytes (SSC-A x FSC-A) was delimited, and from it the gate for single cells (FSC-H x FSC-A) was made. From the resulted population CD3+ followed by CD4+, and CD25+ x Foxp3+ cells were selected resulting in Tregs. Considering Tregs the gates SSC-A x GITR, CD152, LAP and CD134 were made resulting in the positive population for each marker. (TIF) [file pntd.0010105.s004.tif]
